# Ice-shelf retreat drives recent Pine Island Glacier speedup

**DOI:** 10.1126/sciadv.abg3080

**Published:** 2021-06-11

**Authors:** Ian Joughin, Daniel Shapero, Ben Smith, Pierre Dutrieux, Mark Barham

**Affiliations:** 1 Polar Science Center, Applied Physics Lab, University of Washington, 1013 NE 40th Street, Seattle, WA 98105, USA.; 2 British Antarctic Survey, High Cross, Madingley Road, Cambridge CB3 0ET, UK.

## Abstract

Speedup of Pine Island Glacier over the past several decades has made it Antarctica’s largest contributor to sea-level rise. The past speedup is largely due to grounding-line retreat in response to ocean-induced thinning that reduced ice-shelf buttressing. While speeds remained fairly steady from 2009 to late 2017, our Copernicus Sentinel 1A/B–derived velocity data show a >12% speedup over the past 3 years, coincident with a 19-km retreat of the ice shelf. We use an ice-flow model to simulate this loss, finding that accelerated calving can explain the recent speedup, independent of the grounding-line, melt-driven processes responsible for past speedups. If the ice shelf’s rapid retreat continues, it could further destabilize the glacier far sooner than would be expected due to surface- or ocean-melting processes.

## Teaser

Antarctica’s largest contributor to sea level has sped up further as a major section of its floating ice shelf collapsed.

## INTRODUCTION

**Fig. 1:**
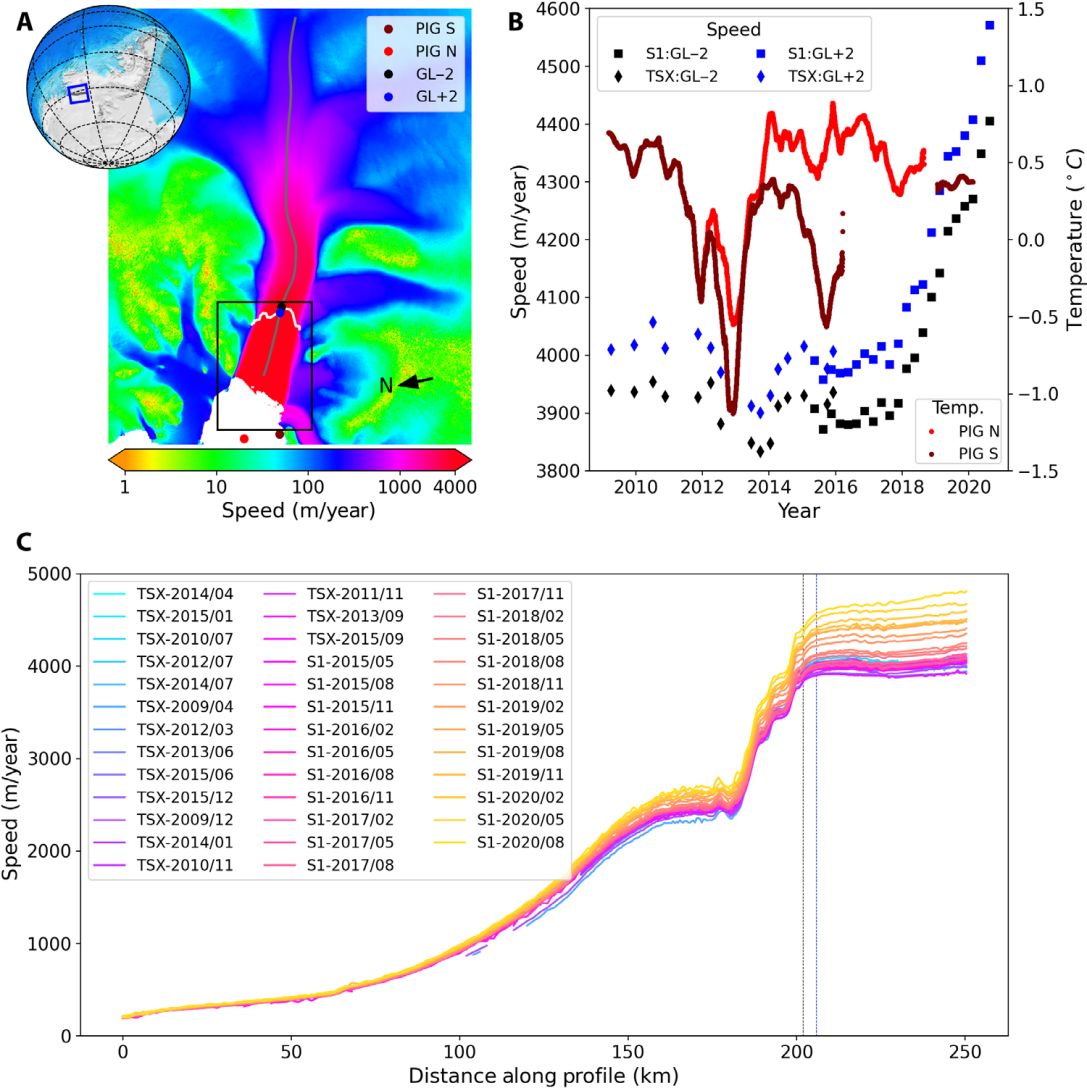
PIG location map and changes in flow speed over the past decade. (**A**) Locations of points where speed is sampled (GL−2 and GL+2), moorings were deployed (PIG N and PIG S), and centerline profile (gray) over a 2019 velocity map of PIG. Black box indicates area shown in Fig. 2. (**B**) Time series of speed at points ~2 km upstream (GL−2) and downstream (GL+2) of the grounding line derived from SAR data collected by the TerraSAR-X/TanDEM-X (TSX) and Copernicus Sentinel 1A/B (S1) missions. The 90-day moving average of mean 450 m-650 m depth ocean temperatures from moorings located toward the north (PIG N) and south (PIG S) ends of the shelf front are shown (*17*, *18*). (**C**) Speeds along centerline profile. Dashed, color-coded lines indicate locations of the GL±2 points.

In the decades since it and neighboring Thwaites Glacier were called “the weak underbelly of the West Antarctic Ice Sheet,” (*1*) Pine Island Glacier (PIG; Fig. 1) has sped up in fits and starts (*2*–*6*) as its grounding line retreated (*7*). In 2009, speeds peaked at ~4000 m/year near the grounding line (where grounded ice first goes afloat) and remained fairly stable over the following 8 years. Several modeling studies have shown that past speedups were due to melt-driven thinning concentrated near the grounding line and the resulting loss of basal traction as the grounding line retreated (*8*–*11*), with ice shelf calving only having a minor influence on ice discharge across the grounding line (*12*). As a result of these speedups, PIG is responsible for more than a quarter of Antarctica’s total sea-level contribution over the past few decades (*13*, *14*).

Beginning in 2015, the Copernicus Sentinel 1A/B synthetic aperture radar (SAR) satellites began collecting data every 12 (before fall 2016) and 6 days (fall 2016 to present) over PIG and much of its catchment. Here, we use these data to investigate changes in PIG’s flow speed as its ice shelf retreated by nearly 20 km since late 2017. While most of the past speedup was due to processes concentrated at the grounding line, earlier work has shown that partial loss of the outer shelf also can cause speedup of the grounded ice (*9*, *15*, *16*). Thus, we analyze changes in flow speed and geometry using an ice-flow model to investigate the cause of the recent speedup.

## RESULTS

We applied standard techniques as described in Materials and Methods to measure flow speed through time from SAR data, as shown in Fig. 1. We selected two points located 2 km upstream (GL−2) and downstream (GL+2) of the nominal grounding line along our reference profile (Fig. 1A), which represents the area most relevant for grounded-ice loss. Figure 1B shows the time series for these points, which indicate relatively steady speeds from 2009 to 2017 with a minor dip around 2013 that has been attributed to reduced oceanic melt during a period with colder water in the ice-shelf cavity (Fig. 1B) (*17*). In late 2017, however, speeds started to increase steadily, leading to a more than 12% increase in near grounding-line speed at the end of the record (September 2020). This recent speedup is also clearly evident in the profile data, extending well inland (>50 km) of the grounding line (Fig. 1C). Over the same time period, moored observations (Fig. 1B) (*18*, *19*) indicate no obvious change in the ocean temperature variability (e.g., increase in heat content) that would trigger melt-driven speedup.

**Fig. 2:**
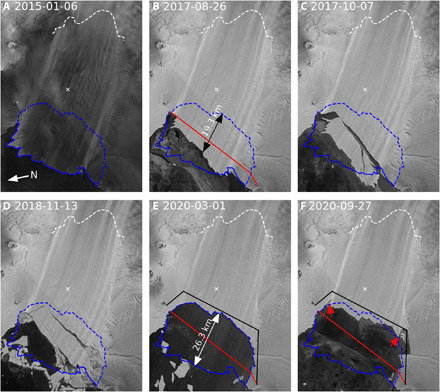
Recent changes in PIG’s ice shelf extent. Sentinel 1A/B images for (**A**) January 2015, (**B**) August 2017, (**C**) October 2017, (**D**) November 2018, (**E**) March 2020, and (**F**) September 2020. The dates are central dates for mosaics created from data collected over 6- or 12-day intervals. The dashed white line shows the grounding line used in the model for the main trunk. For reference, blue lines show the ice front position digitized from the 2015 (solid) and March 2020 (dashed) images. Red (2017) and black (2020) lines show the shelf-front positions used in the model, with the area between them representing the simulated calving event. A white “x” marks the spot where ephemeral grounding has been observed (this grounding does not occur in the simulations) (*24*, *25*). The black and white arrows show the extent of the retreat from January 2015 and August 2017, respectively, to March 2020. The red arrows show areas where the margins lost contact with the sidewalls between March and September 2020.

The Sentinel-1A/B image time series (movie S1) shows a stepped retreat of the central shelf by ~26 km from 2015 to 2020 (Fig. 2E) due to a more than doubling of the steady-state calving rate (~9 versus 4 km/year) over this period. During the early part of the record (January 2015 to August 2017), the shelf retreated by ~7.5 km with no associated speedup. Over this period, much of the area that was lost was from along the shelf’s northern margin where there was little attachment to the grounded ice, and thus, only a minor loss of buttressing seems to have occurred (Fig. 2B). By contrast, the ~19 km of retreat between September 2017 and March 2020 (Fig. 2, B and E) coincides well with the speedup shown in Fig. 1. The calving during this latter period was asymmetric with a much greater loss of contact along the shelf’s southern margin (Fig. 2), leading to an apparent loss of buttressing ability. Although there was a slight readvance (~2.5 km) of the calving front from March to September 2020, speeds continued to increase. During this period, however, the shelf lost additional contact with its margins (see red arrows; Fig. 2F) that likely further reduced its ability to buttress flow.

Although past PIG speedups have been associated with thinning and related grounding-line retreat, the timing of the recent events suggests that the loss of buttressing from the outer shelf is responsible for the recent speed increase. While smaller calving events have not produced substantial variation in speed (*17*), it is likely that this lack of sensitivity was because the ice front had already extended beyond the embayment before calving (e.g., as in 2015–2017 losses) (*16*). Earlier modeling studies of PIG indicate a substantial response in terms of speedup if shelf loss is large enough (*9*, *12*, *15*, *16*). To investigate the cause of the recent speedup, we modeled the 2017–2020 loss (area between red and black lines; Fig. 2F) as a single, instantaneous calving event using a shallow-shelf, ice-flow model of the entire PIG catchment and ice shelf (see Materials and Methods). The simulated calving represents an area loss of 651 km^2^, equivalent to ~20% of the modeled shelf’s initial area.

**Fig. 3:**
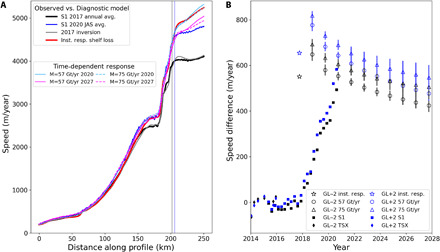
Model and observed response to loss of the outer section of the PIG ice shelf. (**A**) Simulated and observed speeds along the profile shown in Fig. 1. The simulated speeds before and after the instantaneous calving event are shown relative to the observed 2017 and July–September (JAS) 2020 speeds. The simulated speeds in 2020 and 2027 for basal melting of 57 and 75 Gt/year are shown. Color-coded vertical lines indicate locations of the GL±2 points along the profile. (**B**) Observed speed deviations relative to the observed March 2009 to September 2017 average speed. The simulated results are the differences between the models with and without the calving event. The time-dependent simulations show ensemble averages for the 30 random melt functions, with the vertical bars indicating the ±1-σ variation. The date of the simulated shelf removal is September 2017.

Figure 3A shows the simulated instantaneous response to the shelf loss. Near the grounding line, there is good agreement (<100 m/year) with the 2020 observed speed, with greater differences near the shelf front. Following the instantaneous shelf loss, we let the model evolve forward in time over the next decade using shelf-integrated melt values of 57 and 75 gigaton (Gt)/year, each with 30 randomly derived spatial distributions. These melt values roughly bracket the estimated melt of 67 Gt/year for the 2017 model domain (10 Gt/year thinning plus 57 Gt/year net divergence after accounting for surface accumulation). After 3 years, the results remain well matched to the 2020 observations near the grounding line. For the outer shelf, simulated speeds decline thereafter, reaching values that still remain close to the observations after 10 years.

With or without the shelf loss, when the prognostic model starts, there are transients associated with model-data mismatch (fig. S1), which tend to dissipate within a few years. There are additional transients caused by the melt function, which is different than the unknown melt history under which the observed thickness and velocity used to initialize the model evolved. To isolate the response due to the shelf loss, Fig. 3B shows the difference between the reduced-shelf and full-shelf scenarios, which should cancel most common transients due to sources other than shelf loss. For the instantaneous response, the speedup is 552 and 655 m/year for points GL−2 and GL+2, respectively. The corresponding numbers for the peak observed speedup are 494 and 583, which is remarkably good agreement given that we simulated a 3-year stepped retreat as an instantaneous event. There is similarly good agreement with the model after it evolves for 3 years (Fig. 3B). These results are relatively robust (±100 m/year) with respect to variations in both the melt’s spatial distribution and its shelf-integrated total.

As just noted, the speedup shown in Fig. 3 represents a step response to the forcing imposed by the simulated instantaneous calving event. As the simulated results indicate, near the grounding line, there is an instantaneous speedup with a small degree of overshoot in the first year, followed by a gradual decay thereafter. In actuality, there were several discrete calving events staggered with time rather than a single large event, each of which represents a smaller step forcing. Conceptually, we would expect the resulting time-dependent response to be the convolution of the step responses with the individual calving events. Thus, the speedup due to multiple smaller calving events should be similar to that of the one large event, but with a rise time spanning the period over which the events occurred, in good agreement with the observed behavior.

## DISCUSSION

Both the correspondence of shelf loss with the speedup and the agreement between the modeled and observed responses suggest that the recent speedup can be largely attributed to the loss of a large (20%) section of the shelf from 2017 to 2020. This loss of area reduced the ice shelf’s ability to buttress the ice stream by reducing its connection to the embayment’s sidewalls, thus reducing lateral resistive stresses on the shelf. While not at the scale of a complete collapse of the ice shelf (*20*), it is a substantial response to the partial loss. Although through various feedbacks we might expect that the response could grow larger, at least over the next decade, the simulated response indicates that near–grounding line speeds should decline if the shelf front maintains its current position and connection to the embayment’s sidewalls. This slowdown occurs despite simulated grounding line retreat of a few hundred meters, which should increase rather than reduce ice flow. Thus, this decline in speed appears to be a response to the inland migration of thinning, which other models indicate should reduce the slopes near the grounding line, causing slowdown (*11*, *12*). Conversely, it steepens slopes in the interior, causing speedup there (*11*, *12*).

Our model includes damage (e.g., rifting) that weakens the ice-shelf margins (through our inversion for the flow-law coefficient), but this damage does not evolve with time. Given the model-data agreement, post-2017 damage need not be invoked to explain the bulk of the speedup (Fig. 3). Moreover, although there is evidence of increasing damage from 2009 to 2017 (*21*), speeds remained relatively stable (Fig. 1) until the more extensive shelf-front retreat commenced. To the extent that damage may have weakened the margins over this time period, the effect may have been countered by evolving geometry, which can limit speedup near the grounding line (*11*). Thus, in terms of the recent speedup, although there is likely some contribution from damage (*21*), it appears that it plays a secondary role in the 2017–2020 response.

The likelihood of further retreat of PIG’s ice shelf in the coming years to decades remains unclear. Although surface melt events occasionally occur, there is no evidence of melt ponds causing hydrofractures that would have contributed to the recent calving (movie S1). Furthermore, climate models indicate that these conditions are not likely to occur in this century for PIG or many other Antarctica glaciers (*22*). Instead, the accelerated calving appears to be due to large rifts that form well upstream of the calving front, which have been attributed to enhanced rifting and loss of ice along the northern shear margin (*23*). Alternatively, ~10 km upstream of the current ice front (see Fig. 2), there is a spot that ephemerally grounds (*24*) as deep ice-shelf keels migrate over a bathymetric high (*25*). Examination of the Sentinel 1A/B time series suggests that at least some of these rifts originate just to the south of this feature. These nascent rifts are too indistinct in the SAR imagery at this stage of their formation, however, to draw firm conclusions as to whether the grounded spot contributes to their formation. Last, there was extensive rifting along the southern margin in the area that retreated (Fig. 2), which may also have contributed to the excess calving.

Irrespective of the exact cause, recently initiated rifting processes appear to have driven much of the ice-front retreat via calving at a nonsteady rate (*23*). After the recent retreat, the shelf front is potentially now in a more sheltered/confined part of the embayment, which may slow or stop the enhanced rifting. Rifts appear to be less prevalent in the central shelf in the latter part of the 2020 Sentinel 1A/B record (movie S1). Over the same period, however, there is substantial rifting along the margins that could lead to further loss of buttressing and retreat over the next few years. Thus, the long-term stability of PIG appears to depend heavily on how calving rates affect the viability of its remaining ice shelf (*16*, *20*). A stabilization of the ice front near its current position is possible, but so is a retreat far more rapid than expected from ocean- and surface melt–related processes alone. The extent to which these rifting processes might influence other ice shelves remains unclear, leading to increased grounded-ice discharge. Given that these processes could cause ice-shelf loss earlier than expected from surface melt forcing (*22*), further study and observation is warranted.

## MATERIALS AND METHODS

Our results depend on remote-sensing observations and numerical models as described below.

### Ice-flow velocity and SAR observations

We used standard speckle-tracking methods applied to Copernicus Sentinel 1A/B data to produce a time series of velocity change for much of the PIG basin from 2015 to September 2020 (*26*). The high accumulation rates in this region cause image pairs to decorrelate rapidly, so individual speckling-tracking results can be noisy and subject to large gaps. As a result, the data are aggregated to produce quarterly (3-month) estimates by weighted averaging, where the relative weights are proportional to the inverse squared errors of their respective individual estimates (*26*). In addition, at each location in the final map, a variable number of points are averaged because, to a greater or less extent, all individual velocity maps have gaps where a successful estimate could not be achieved. As such, the actual central date of the quarterly estimate at each point may be skewed differently from the nominal central date (*27*). Here, we assume that these deviations are small and plot the results relative to the nominal central date.

The S1A/B data extend an existing time series from 2009 to 2015 created using data from the TerraSAR-X and TanDEM-X missions (collectively referred to as TSX) (*11*, *17*). These data are irregularly sampled in time and averaged with inverse-error weighting. The nominal central date is determined as the day midway between the first and last images used in the estimate (2 to 5 months). We also geocoded the sequence of Sentinel 1A/B SAR imagery over PIG to produce a record of ice-shelf retreat (movie S1).

### Ice-sheet model

We use an open-source, shallow-shelf, ice-sheet model, icepack, to simulate the response of PIG to a partial loss of the ice shelf (*28*). The model uses an unstructured finite-element mesh, with continuous Galerkin elements of degree 2. Resolution is variable with the finest resolution of ~300 m concentrated on the fast-moving trunk and grounding line. The underlying solvers for both the diagnostic and prognostic equations are based on the Firedrake finite-element solver package (*29*).

We use an inverse solver with Tikhonov regularization to determine basal shear stress for a regularized Coulomb friction model constrained by the 2017 observations used in an earlier study (*11*). The modeled 2017 front (Fig. 2) is representative of mid-2017 conditions, but it is set back a few kilometers on the south side to avoid gaps in the 2017 velocity map used to initialize the model. Regularized Coulomb friction is implemented slightly differently but is functionally similar with a transition from Weertman-like to Coulomb-like behavior at around 300 m/year. On the shelf, we solve for the flow-law parameter, *B* (*9*), which implicitly includes the effect of damage. The model was constrained largely with an elevation dataset used in earlier work (*11*), which was derived from airborne and spaceborne altimetry data and optical stereo imagery. The grounding line evolves freely with a point remaining grounded as long as its elevation remains above flotation.

To simulate calving, we created a new mesh with a shelf front based nominally on March 2020 conditions (Fig. 2). We then interpolated the 2017 solutions for friction and flow-law coefficients to this mesh, which in effect simulates the instantaneous loss of the outer part of the shelf. To examine the time-dependent response, we simulated the evolution of the system for the decade after the initial shelf loss. In the time-dependent model, we use depth-dependent parameterized melt functions similar to other studies (*9*, *10*). To better understand the sensitivity to the choice of melt function, we ran each simulation with 30 realizations of randomly generated melt-rate parameterizations. Qualitatively, these functions are similar in that they tend to concentrate melt at depth, but they produce substantial variation in spatial distribution. Statistically, half of the melt functions produce peak melt at the grounding line, while the other half produce peak melt a few kilometers from the grounding line as more detailed models suggest (*30*). For all melt parametrizations, the melt rate at each step is normalized to produce the same shelf-integrated melt (57 or 75 Gt/year). Results from each set of 30 realizations are averaged, and the standard deviation is used to illustrate the sensitivity to an individual parameterization.

The model was constrained with the average velocity field from 2017 Sentinel 1A/B data, with coverage gaps in the upper catchment filled with data from the MEaSUREs Version 2 velocity map of Antarctica (*31*). We used the bed data from the BedMachine Antarctica Version 2, which we adjusted near the grounding line to force the grounding to be consistent with the 2017 surface using the procedure described in an earlier study (*11*). The surface mass balance data used in the prognostic model were derived from airborne radar observations (*6*). Table S1 provides links to the datasets and open-source model code needed to reproduce the results, including the speed data presented in Figs. 1 and 3.

### Calving front retreat

From January 2015 to March 2020, the central part of PIG’s ice-shelf front retreated by 26.3 km (Fig. 2). Ignoring the recent speedup, we assume a nominal shelf-front speed of 4000 m/year (Fig. 1), which implies that the steady-state (with respect to mean front position) calving rate would need to be 4 km/year. If there had been no calving, the glacier would have advanced by 20.6 km over this 5.2-year period. Thus, the calving rate necessary to match the observed 26.3-km retreat is 9.1 km/year. Care must be exercised in interpreting these short-term calving rates, as a sustained readvance or further retreat could produce a substantially different long-term rate (e.g., the rate from 2017 to 2020 exceeds 11 km/year). Nonetheless, the recent half-decade average calving rate of ~9 km/year has caused the ice front to retreat to a position of minimum extent in the satellite era (*32*).

### Moored observations

Mooring observations in front of PIG started early 2009 (*18*) and were maintained since by a series of programs. Temperature loggers consistently observed the depth range between 450 and 650 m at the PIG S and PIG N sites (Fig. 1A), thereby providing a sense of the thermocline variability in front of the ice shelf, serving as good proxies for heat content variability and associated ice-shelf melt (*19*).

## Supplementary Materials

Supplementary material for this article is available at http://advances.sciencemag.org/cgi/content/full/7/24/eabg3080/DC1
